# Suicide Risk in People with Hearing Impairment in the Post-COVID-19 Period: The CaViDAuCo Study

**DOI:** 10.3390/jcm14093130

**Published:** 2025-04-30

**Authors:** Nerea Moreno-Herraiz, Iván Cavero-Redondo, Iris Otero-Luis, Carlos Pascual-Morena, María Dolores Gómez-Guijarro, Irene Martínez-García, Alicia Saz-Lara

**Affiliations:** 1 Carvascare Research Group, University of Castilla-La Mancha, 16071 Cuenca, Spain; nerea.moreno@uclm.es (N.M.-H.); iris.otero@uclm.es (I.O.-L.); doloresgg18@gmail.com (M.D.G.-G.); irene.mgarcia@uclm.es (I.M.-G.); alicia.delsaz@uclm.es (A.S.-L.); 2 Health and Social Research Center, University of Castilla-La Mancha, 16071 Cuenca, Spain; carlos.pascual@uclm.es; 3 Faculty of Nursing, University of Castilla-La Mancha, 02071 Albacete, Spain

**Keywords:** COVID-19 pandemic, hearing impairment population, suicide risk, quality of life, mental disorders

## Abstract

**Background/Objectives**: During the COVID-19 pandemic, suicide risk increased in the general population and persisted in the post-pandemic period. People with hearing impairment faced communication barriers that negatively affected their mental health. However, there is no evidence on whether they have an increased suicide risk in the post-pandemic period. This study aimed to assess the association between mental disorders, quality of life, and suicide risk in individuals with hearing impairment in the post-COVID-19 period. **Methods**: A cross-sectional study was conducted with 103 participants with hearing impairment from the CaViDAuCo study. Adjusted and unadjusted differences in mental disorders (depression, anxiety, and stress) and quality of life (physical and mental) were analyzed using Student’s *t* test and ANCOVA according to suicide risk. **Results**: Depression, anxiety, stress, and mental quality of life in people with hearing impairment were significantly associated with suicide risk (unadjusted, models 1 and 2, *p* < 0.001; Cohen’s *d* = 1.4, 1.4, 1.3, and −1.0, respectively). Due to the cross-sectional design, no causal relationships can be established. **Conclusions**: In the post-pandemic period, participants with hearing impairment exhibited a significant association between suicide risk, mental disorders, and poor mental quality of life. Although causality cannot be established, and the results should be interpreted with caution due to the small sample size, these findings underscore the need to improve mental health accessibility and implement inclusive communication policies. Further research is needed to better understand these associations and design effective interventions that promote the mental health and quality of life of people with hearing impairment.

## 1. Introduction

In December 2019, a sudden outbreak of severe infection associated with a new severe acute respiratory syndrome coronavirus (COVID-19) occurred in Wuhan, China, and rapidly spread worldwide, becoming a global pandemic [1,2]. Measures taken to prevent the spread of COVID-19 included house confinement, mobility restrictions, hand hygiene and hygienic measures, social distancing, and mandatory use of masks [3], but these measures caused psychological distress and had a major impact on the psychosocial well-being of the general population [4]. However, despite adopting these preventive measures, according to the Spanish National Institute of Statistics, deaths due to COVID-19 in 2020 totaled 33,312, and in 2021, they reached 29,300 [5].

These restrictions, particularly for people with hearing impairment (HI), have led to additional isolation and decreased accessibility in terms of social interaction, communication, and access to COVID-19-related information and resources, could contribute to poorer personal and emotional well-being [6,7]. Online activities and communications via video calls have increased, harming this population [4,8]. Face-covering mandatory hygiene masks have a major impact on communication, limiting facial expressions and preventing lip reading, not to mention being a major communication barrier if healthcare workers are wearing personal protective equipment (PPE) [6,8,9]. In addition, companions are restricted during healthcare, and it is highly important for people with HI to be accompanied by a family member or a sign language interpreter (SLI) to receive quality care and effective communication [8,10]. Finally, during the COVID-19 pandemic, continuous broadcasts of adequate and reliable information were inaccessible due to the lack of adaptations, such as the absence of SLIs and subtitles and the impossibility of accessing lip reading [8].

Concerning the COVID-19 pandemic itself, several previous studies have explored mental disorders and quality of life (QoL) in people with HI. The most frequently assessed mental disorders in this population were anxiety [11,12,13,14,15,16], depression [11,13,14,15,17], and stress [11,14,17]. According to these studies, people with HI suffered worse mental health than people without HI during the pandemic due to increased communication barriers, lack of access to information, and an increase in the number of mental disorders they already suffer from because HI is considered a risk factor for mental disorders [4,6,18].

Suicide, a worldwide public health problem, is associated with mental disorders according to the World Health Organization (WHO), and this connection is associated with an incidence of death by suicide every 40 s [19]. According to the Spanish National Institute of Statistics, there were 3941 deaths by suicide in Spain in 2020, an increase of 7.4% compared with 2019 [19]. However, the statistics only reflect deaths, not suicide attempts or suicidal ideation, which is estimated to be 20 times more frequent [20]. There are a number of possible mechanisms that explain how the suicide risk increases when people experience frustrated belongingness and an increased perceived burden due to repeated exposure to painful or fearful experiences [21]. These factors may be especially relevant for people with HI due to communication barriers, social isolation, limited access to information, and mental health resources [6,7], and they may exacerbate emotional distress, thereby increasing suicidal behaviors and ideation [6,7,21]. Another possible explanation is that people with HI are at greater risk of abuse due to persistent communication barriers, which limit their ability to seek help or report interpersonal violence [22,23]. Both physical and emotional abuse and neglect contribute to poor mental health among people with HI and may be associated with self-injurious thoughts and behaviors [23,24].

There is evidence that there was an increase in consultations for suicide attempts and suicidal ideation in the post-pandemic period [19], with a significant increase in the ratio of suicidal ideation to suicide attempts [20]. During the pandemic period, there were increases in symptoms of depression, anxiety, and insomnia in the general population and in people with HI in particular; these conditions have been observed alongside increased suicide risk [19], and suicidal ideation appeared to coincide with adjustment problems and loneliness. Although there is evidence of an increase in short-term mental disorders during the pandemic in people with HI, it is still necessary to investigate whether there have been any changes in the suicide risk of this population over the long-term.

Therefore, the aims of this study were as follows: (1) to assess the associations between mental disorders (depression, anxiety, and stress) and suicide risk in people with HI in the post-COVID-19 period; and (2) to assess the associations between quality of life—both physical and mental—and suicide risk in people with HI in the post-COVID-19 period.

## 2. Materials and Methods

### 2.1. Study Design, Participants, and Sample Size

The cross-sectional CaViDAuCo study was conducted in different centers and associations of people with HIs in Spain to analyze variables collected at a single point in time. Recruitment of participants was carried out at the University of Castilla-La Mancha, Cuenca, by the research staff (including physicians and nurses), who contacted a total of 57 centers and associations of people with HI in Spain, including ASPAS, APSORGU, APROSOJA, FASICAN, FESCAN, FEXAS, and FESORMANCHA, among others (Table S1). A convenience sampling method was used through an open call by email. Research staff sent both the questionnaires and informed consent forms by email. Participation was completely voluntary. The sample size was calculated a priori using Epidat 4.2 software based on an estimated large effect size (Cohen’s *d* = 1.0) obtained from a previous study using the Overall Depression Severity and Impairment Scale [25]. This scale was selected exclusively for the purpose of estimating the sample size, as no prior studies assessing the association between mental disorders and suicide risk in populations with HIs were available at the time of study design. The Overall Depression Severity and Impairment Scale was chosen because of its conceptual similarity, as it assesses the severity of depression, which is a well-established predictor of suicidal behavior. This proxy approach allowed us to ensure a robust sample size to detect differences relevant to our primary hypothesis regarding the association between mental disorders and suicide risk. By converting the effect size to Cohen’s *d*, we ensured a standardized and scale-independent estimate, which allows for valid extrapolation even when a different instrument was ultimately used in the study. However, we acknowledge that the use of a large effect size (Cohen’s *d* = 1.0) in the sample size calculation may be optimistic and could inflate power estimates. Future studies should aim to use more conservative and empirically derived effect sizes as more data become available. Patients who met the inclusion and exclusion criteria were invited to participate in the study, and 103 participants were eventually enrolled between 1 March 2023 and 1 October 2023. This study followed the Strengthening the Reporting of Observational Studies in Epidemiology (STROBE) statement [26]. The eligibility criteria for individuals were as follows: (1) hearing impaired at birth or later; (2) over 18 years of age; and (3) used sign language, spoken language, or both. The exclusion criteria for individuals were as follows: (1) participated in another study; (2) provided no written informed consent; and (3) prior diagnosis of psychiatric pathology.

### 2.2. Ethical Considerations

The research protocol for this study was approved by the Clinical Research Ethics Committee of the Cuenca Health Area (REG: 2022/PI3322). Written informed consent to participate was obtained from all the subjects involved in the study.

### 2.3. Variables

The variables analyzed were collected via online self-administered questionnaires.

#### 2.3.1. Main Variables

QoL (both physical and mental) was assessed using the SF12 health questionnaire. The subjects answered 12 questions about what they thought of their health and to what extent they were able to perform usual activities [27]. Once the answers were obtained, they were individually analyzed through the SF12 OrthoToolKit calculator [28], which calculates the physical and mental level scores for each subject (Table S2).

Depression, anxiety, and stress were assessed using the DASS-21 scale. The subjects responded to 21 questions on the scale, with four numerically ranked responses associated with the degree to which the statements had occurred during the past week. After the responses were obtained, item scores (subscales) for depression (items 3, 5, 10, 13, 16, 17, and 21), anxiety (items 2, 4, 7, 9, 15, 19, and 20), and stress (items 1, 6, 8, 11, 12, 14, and 18) were interpreted individually. For each subscale, the scores of the corresponding items are summed, and the result is then multiplied by two. The final total score was classified according to the following cut-off points to assess the degree of symptomatology: depression: normal (0–9), mild (10–13), moderate (14–20), severe (21–27), and extremely severe (28+); anxiety: normal (0–7), mild (8–9), moderate (10–14), severe (15–19), and extremely severe (20+); and stress: normal (0–14), mild (15–18), moderate (19–25), severe (26–33), and extremely severe (34+) [29] (Table S3).

Plutchik’s Suicide Risk Scale. The subjects responded to the 15 questions of the scale only with affirmative or negative answers. The results were interpreted individually, with each affirmative response receiving 1 point and a negative response receiving 0 points, for a total possible score between 0 and 15 points. As the scale score increases, so does the suicide risk, establishing a cut-off point greater than or equal to 6 to consider the suicide risk to be significant [30] (Table S4).

#### 2.3.2. Covariates

The self-reported covariates included in this study were age, sex, whether people were deaf from birth, hearing aids used, and the language used (sign language, spoken language, or both).

### 2.4. Statistical Analysis

Normal probability plots and the Kolmogorov–Smirnov test were used to verify the normality of the distribution of continuous variables. Descriptive data for the total sample are shown as the means and standard deviations (SDs) or proportions (%), as appropriate. The chi-square statistic was used to evaluate the prevalence of people at risk or not at risk of suicide according to the following categories: depression (mild, moderate, severe, or extremely severe), anxiety (mild, moderate, severe, or extremely severe), and stress (mild, moderate, severe, or extremely severe). In addition, the mean values and SDs were calculated for mental disorders (depression, anxiety and stress) and QoL (both physical and mental) according to suicide risk. Student’s *t* test for independent samples and analysis of covariance (ANCOVA) were performed for the total sample between mental disorders and QoL and suicide risk. In addition, ANCOVA was performed adjusting for all variables, i.e., deaf at birth or later, hearing aids used and language used (sign language, spoken language, or both) as categorical covariates and age as a continuous covariate. Finally, we calculated Cohen’s *d* for the effect size, considering the effect to be trivial (<0.2), small (0.2–0.5), medium (0.5–0.8), or large (>0.8) [31]. All analyses were performed for the overall population and by sex. All the statistical analyses were performed using IBM SPSS 28 (SPSS Inc., Chicago, IL, USA), and *p* < 0.05 was considered statistically significant.

## 3. Results

### 3.1. Characteristics of the Study Population (In the Post-COVID-19 Period)

The sample size previously calculated using Epidat software indicated that 37 participants would be needed to detect an estimated effect size (Cohen’s d) of 1. Ultimately, the CaViDAuCo study included a total of 103 participants with HI aged between 19 and 74 years (mean age 35.4 ± 12.1 years). Among the total sample, 57 (55.3%) were women, and 46 (44.7%) were men. In addition, 64.1% were HIs from birth, and 45.6% used both sign language and spoken language. Table 1 shows the baseline characteristics of the included participants.

### 3.2. Prevalence of Mental Disorders (Depression, Anxiety, and Stress) According to Suicide Risk

Table 2 shows the prevalence of depression, anxiety, and stress according to categories (mild, moderate, severe, and extremely severe) in people with HI in the post-COVID-19 period according to suicide risk. Three years after the COVID-19 pandemic, the presence of mental disorders remained significantly associated with suicide risk. The prevalence of depression, anxiety, and stress was significantly higher among people at risk of suicide compared to those not at risk, both in the total population and in men and women. Specifically, depression, anxiety, and stress were more prevalent in the total population (*p* < 0.001), in men (*p* = 0.002, *p* = 0.006, and *p* = 0.024, respectively), and in women (*p* < 0.001, *p* < 0.001, and *p* = 0.007, respectively). Notably, men showed higher rates of extremely severe depression (33.3% vs. 15.4%) and extremely severe anxiety (44.4% vs. 15.4%) compared to women, as well as a higher prevalence of severe stress (33.3%). In contrast, women had higher rates of moderate depression (53.5% vs. 33.3%), severe anxiety (30.8% vs. 11.1%), and extremely severe stress (23.1%).

### 3.3. Associations of Mental Disorders (Depression, Anxiety, and Stress) and Quality of Life (Physical and Mental) with Suicide Risk

Table 3 shows the results of the mean differences in mental disorders and QoL according to suicide risk in the total population. In the post-pandemic period, people at suicide risk had higher values of depression, anxiety, and stress (9.6 ± 4.7, 7.5 ± 5.3, and 10.6 ± 4.7, respectively) than did people not at suicide risk (3.9 ± 3.8, 2.6 ± 3.0, and 5.2 ± 4.2, respectively) (for all variables, unadjusted model and models 1 and 2, *p* < 0.001). In addition, people at suicide risk had lower mental quality of life (38.6 ± 9.0) than people who were not at suicide risk (47.7 ± 9.5) (unadjusted model and models 1 and 2, *p* < 0.001).

Table 4 shows the results of the mean differences in mental disorders and QoL according to suicide risk in both men and women in the post-pandemic period. In men, those at suicide risk had higher values for depression, anxiety, and stress (10.1 ± 4.2, 7.7 ± 4.5, and 9.9 ± 3.9, respectively) than those who were not at suicide risk (3.5 ± 3.1, 2.4 ± 2.4, and 4.9 ± 4.5, respectively) (for depression and anxiety, unadjusted model and models 1 and 2, *p* < 0.001; for stress, unadjusted model, *p* = 0.004; model 1, *p* = 0.006; and model 2, *p* = 0.011). Similarly, women at suicide risk had higher values of depression, anxiety, and stress (9.1 ± 5.2, 7.4 ± 5.9, and 11.0 ± 5.3, respectively) than did those who were not at suicide risk (4.1 ± 4.4, 2.7 ± 3.4, and 5.4 ± 3.9, respectively) (for depression, unadjusted model, *p* < 0.001; model 1, *p* = 0.002; and model 2, *p* = 0.003; for anxiety and stress, unadjusted model, models 1 and 2, *p* < 0.001). In addition, people at suicide risk had lower mental quality of life (36.1 ± 8.4) than people who were not at suicide risk (47.5 ± 9.7) (unadjusted model and model 1, *p* < 0.001; and model 2, *p* = 0.001).

## 4. Discussion

### 4.1. Main Findings

To our knowledge, this is the first study to evaluate the associations between mental disorders (such as depression, anxiety and stress) and QoL (both physical and mental) and suicide risk in a population with HI in the post-pandemic period. Our findings showed that in the post-pandemic period, depression (large effect), anxiety (large effect), stress (large effect) and mental QoL (trivial effect) could be positively associated with suicide risk, with higher scores than in individuals not at suicide risk in the population with HI. However, due to the lack of pre-pandemic data in people with HI, we cannot determine whether these risk levels have changed as a consequence of the pandemic.

Mental disorders and QoL during and during the post-COVID-19 period.

During the COVID-19 period, both the general population without HI [32,33,34] and those with HI experienced increased levels of mental disorders such as depression, anxiety and stress [10,11,12,14,16]. Similarly, physical and mental QoL declined in the general population [32], while people with HI reported even poorer QoL [35,36,37]. After 12 months, depression in the general population was observed, with a mild worsening trend and some individuals experiencing persistent or fluctuating psychological distress [38]. Psychological effects, such as anxiety, depression, and stress, may worsen over time and lead to long-term sequelae [38,39,40].

### 4.2. Mental Disorders in the Post-COVID-19 Period in the Population with Hearing Impairment

During the COVID-19 pandemic the mental health of people with HI has been affected, mental disorders have been observed such as depression, anxiety, and stress [6,11,12,13,15,17]. However, no prior evidence exists on the prevalence of these mental disorders in the post-pandemic period for this population. In the general population, a worsening mental disorders was observed during the pandemic period, and these have been identified as potential risk factors [41]. Suicide rates increased after the confinement period [42], and mental health issues and suicidal behaviors are expected to peak in the post-pandemic period [41], likely affecting specific groups such as people with HI. This population tends to have poorer mental health due to their disability [4,6,18,43] and may be at higher suicide risk than those without HI before the pandemic [43,44,45,46,47]. During the pandemic period, it has been observed that people with HI may be at higher suicide risk [48].

### 4.3. QoL in the Post-COVID-19 Period in the Population with Hearing Impairment

With respect to QoL, a decline in both physical and mental QoL was observed in the general population without HI during the lockdown period [32]. Following the lifting of restrictions, physical activity appeared to return to normal levels; however, a continued decline in mental activity was noted in the post-pandemic period [32]. While HI has been linked to lower QoL [43], studies have reported poorer QoL in people with HI during the pandemic period [35,37]. However, evidence of long-term changes in QoL following the end of restrictions, or in the absence of the pandemic, remains limited.

### 4.4. Barriers Faced by People with Hearing Impairment During the COVID-19 Period

The poorer mental health and mental QoL of people with HI may stem from a lack of coping skills and emotional awareness. Factors such as government measures, family loss, job disruptions, poverty, and psychological adversities during the pandemic may have contributed to this [49,50]. In addition, increased demand for mental health services has led to limitations, creating significant care gaps. Face-to-face care was often unavailable, shifting to remote interventions, which posed challenges for people with HI due to the lack of subtitles, SLI and the impossibility of lip reading [7,41,51]. Communication barriers were further exacerbated by PPE use and telecare [4,6,7], while masks also reduced perceived empathy, making communication even more difficult for people with HI [8,9].

Deficiencies in psychiatric emergency services, including suicide prevention [51], have been reported due to high demand and a shortage of mental health staff [52]. During the post-pandemic period, our findings showed that depression, anxiety, stress, and poor mental QoL in people with HI were observed alongside indicators of increased suicide risk. Therefore, it is crucial to improve online mental health resources and interventions for the overall population [41], particularly for individuals with HI, to ensure better accessibility through specialized applications and adaptations, such as written materials, images and videos, vibrations, colored lights, audio-to-text converters, and SLI [14,53,54,55].

In addition, most health professionals lack the necessary skills to communicate effectively with people with HI, causing stress and insecurity for both parties. It is essential that they understand the communication needs of people with HI and gain better insight into the deaf community [54,55]. For individuals with HI, support during healthcare is very important for optimal care and effective communication. However, during the pandemic, restrictions on accompanying persons, such as SLIs or family members, led to a lack of communication support for deaf individuals and for hard-of-hearing individuals [8,10].

During the COVID-19 period, irresponsible media coverage of suicide and repeated reports about the pandemic may have increased suicide risk and self-harm, especially among young people [41,51]. The prevalence of suicide is greater in individuals with previous suicide attempts, and self-harm ideations are more frequent than suicide attempts [20,42]. Many people avoid seeking mental health services due to stigma, often delaying care for 8–15 years [52]. In addition to mental disorders such as depression, anxiety, and stress, social isolation and loneliness could contribute to suicide risk, highlighting the need for support for those living alone [41]. Evidence suggests that people with HI experienced increased loneliness and isolation during the pandemic, with those living alone suffering more mental disorders than those living with others [14]. Additionally, poorer hearing ability has been linked to higher levels of loneliness [15].

Finally, studies [49,50] have shown that people with conditions that can contribute significantly to mental health are at increased risk of persistent psychological sequelae. HI is considered a risk factor for mental disorders [4,6,18]. For all the reasons mentioned above, mental disorders and poorer mental QoL in people with HI may persist in the post-pandemic period and could be associated with suicide risk, as suggested by our study.

In addition to the aforementioned psychosocial barriers, future research should also examine the impact of interpersonal factors such as abuse and neglect, which are often overlooked among people with HI due to persistent communication barriers. These experiences may exacerbate emotional distress and could be associated with increased suicide risk. Understanding these dynamics is crucial to developing more comprehensive suicide prevention strategies tailored to the specific vulnerabilities of this population and contributing positively to their mental health [22,23,24].

### 4.5. Limitations

This study has several limitations that should be considered. First, the sample size of people with HI was small (103 participants). Furthermore, since convenience sampling was used, there is a possibility of self-selection bias, which limits the representativeness of the sample and may influence the generalizability of the results. Second, although this was a study aimed at the population with HI, we must consider whether the questionnaires adapted for sign language were understandable and whether they were understood correctly, depending on the comprehension of this population. In addition, the questionnaires were self-reported online, which may influence the subjectivity of the participants. Third, due to the nature of the study design, a causal association cannot be established. Fourth, we did not assess the cause of HI, and this study may be biased toward people with HI at birth or later, so our results should be interpreted with caution. Fifth, the severity of each participant’s disability was not assessed, only whether deafness was present at birth or acquired later and, if acquired, their age. Sixth, this study did not consider other covariates, such as other types of disability (visual, sensory, cognitive, etc.), ethnicity, comorbid medical diagnoses (such as diabetes, hypertension, etc.), and whether participants were taking medication. Seventh, although the sample size calculation was based on the Overall Depression Severity and Impairment Scale due to the lack of previous data in similar populations from the DASS-21, this scale was not used for data collection in our study. This could introduce slight discrepancies between the estimated and observed effect sizes, although the use of standardized metrics such as Cohen’s *d* mitigates this limitation. Eighth, more superior suicide risk rating scales, such as the Columbia-Suicide Severity Rating Scale, the Beck Scale for Suicide Ideation, or the Suicide Behaviors Questionnaire—Revised, were not used; however, Plutchik’s Suicide Risk Scale was used because in a population with HI, it can be flexibly adjusted or administered, as these participants may have difficulty expressing their emotions verbally due to limited access to spoken language. This scale allows for a more individualized approach than other more rigid scales. Ninth, the large effect sizes observed (e.g., Cohen’s d >1.4) when comparing participants with and without suicide risk should be interpreted with caution. These values may reflect low within-group variability or small group sizes, especially given the limited number of participants at suicide risk (*n* = 22), rather than true population-level differences. This small sample may have increased the likelihood of statistical fluctuation and inflated effect size estimates. Therefore, it is necessary to repeat the study with larger, more representative samples to confirm the magnitude of these associations. Furthermore, longitudinal studies with larger sample sizes and in different population types (in terms of age, gender, pathologies, etc.) are needed to further consolidate these findings.

## 5. Conclusions

In the post-pandemic period, people with HI continue to experience mental disorders, such as depression, anxiety, and stress, and have poor mental QoL. Given that mental disorders and poor mental QoL are risk factors for suicide, our findings indicate a significant association, rather than a causal relationship, between these factors and suicide risk in participants with HI. However, our results are based on a small sample size; therefore, they should be interpreted with caution and not generalized to the general population. It is essential to improve accessibility in mental health services by ensuring appropriate adaptations for people with HI, such as captioning, SLIs, images, and videos, among others. Additionally, raising awareness and implementing regional and national policies to improve communication tools can help address their specific needs and reduce the suicide risk. Further research is needed to better understand these associations and to design effective interventions that promote mental health and QoL in people with HI, helping reduce suicide risk.

## Figures and Tables

**Table 1:** Characteristics of the study participants.

Variable	Total (*n* = 103)	Men (*n* = 46)	Women (*n* = 57)
Age (years)	35.4 ± 12.1	34.7 ± 11.0	36.0 ± 12.9
Hearing impairment			
From birth	66 (64.1%)	34 (73.9%)	32 (56.1%)
Later	37 (35.9%)	12 (26.1%)	25 (43.9%)
Hearing aids			
Cochlear implants	27 (26.2%)	12 (26.1%)	15 (26.3%)
Hearing aids	42 (40.8%)	19 (41.3%)	23 (40.4%)
Both	13 (12.6%)	6 (13.0%)	7 (12.3%)
None	21 (20.4%)	9 (19.6%)	12 (21.1%)
Languages used			
Sign language	18 (17.5%)	9 (19.6%)	9 (15.8%)
Spoken language	38 (36.9%)	16 (34.8%)	22 (38.6%)
Both	47 (45.6%)	21 (45.7%)	26 (45.6%)
Mental disorders			
Depression	5.1 ± 4.7	4.8 ± 4.2	5.3 ± 5.0
Anxiety	3.6 ± 4.1	3.4 ± 3.6	3.8 ± 4.5
Stress	6.3 ± 4.8	5.9 ± 4.7	6.7 ± 4.8
Quality of life			
Physical QoL	52.2 ± 8.2	52.7 ± 7.8	51.8 ± 8.5
Mental QoL	45.7 ± 10.1	46.8 ± 9.6	44.9 ± 10.5

The data are shown as the mean ± standard deviation (SD) or number of subjects (percentage), *n* (%).

**Table 2:** Prevalence of mental disorders (depression, anxiety, and stress) according to suicide risk in the post-COVID-19 period.

	Total					Men					Women				
Variables	*n*	Suicide Risk	*n*	No Suicide Risk	*p*	*n*	Suicide Risk	*n*	No Suicide Risk	*p*	*n*	Suicide Risk	*n*	No Suicide Risk	*p*
Depression	22	81.8%	81	33.3%	<0.001	9	77.7%	37	27.0%	0.002	13	84.6%	44	38.6%	<0.001
Mild	1	4.5%	12	14.8%		0	0.0%	1	2.7%		1	7.7%	11	25.0%	
Moderate	10	45.5%	11	13.6%		3	33.3%	8	21.6%		7	53.5%	3	6.8%	
Severe	2	9.1%	3	3.7%		1	11.1%	1	2.7%		1	7.7%	2	4.5%	
Extremely severe	5	22.7%	1	1.2%		3	33.3%	0	0.0%		2	15.4%	1	2.3%	
Anxiety	22	72.7%	81	27.2%	<0.001	9	66.6%	37	21.6%	0.006	13	77.0%	44	31.8%	<0.001
Mild	3	13.6%	10	12.3%		1	11.1%	3	8.1%		2	15.4%	7	15.9%	
Moderate	2	9.1%	8	9.9%		0	0.0%	2	5.4%		2	15.4%	6	13.6%	
Severe	5	22.7%	2	2.5%		1	11.1%	2	5.4%		4	30.8%	0	0.0%	
Extremely severe	6	27.3%	2	2.5%		4	44.4%	1	2.7%		2	15.4%	1	2.3%	
Stress	22	59.0%	81	19.8%	<0.001	9	66.6%	37	21.6%	0.024	13	53.9%	44	18.2%	0.007
Mild	2	9.1%	8	9.9%		2	22.2%	4	10.8%		0	0.0%	4	9.1%	
Moderate	3	13.6%	4	4.9%		1	11.1%	2	5.4%		2	15.4%	2	4.5%	
Severe	5	22.7%	2	2.5%		3	33.3%	1	2.7%		2	15.4%	1	2.3%	
Extremely severe	3	13.6%	2	2.5%		0	0.0%	1	2.7%		3	23.1%	1	2.3%	

The data are shown as the number of subjects and percentage.

**Table 3:** Unadjusted and adjusted differences in mental disorders (depression, anxiety, and stress) and quality of life (physical and mental) according to suicide risk in the post-COVID-19 period, analyzed using Student’s *t* test and ANCOVA in the total population.

		Total				
Variable	Suicide Risk (*n* = 22)	No Suicide Risk (*n* = 79)	Cohen’s *d* (95% CIs)	Unadjusted Model	Model 1	Model 2
Depression	9.6 ± 4.7	3.9 ± 3.8	1.4 (0.9, 1.9)	<0.001	<0.001	<0.001
Anxiety	7.5 ± 5.3	2.6 ± 3.0	1.4 (0.8, 1.9)	<0.001	<0.001	<0.001
Stress	10.6 ± 4.7	5.2 ± 4.2	1.3 (0.7, 1.8)	<0.001	<0.001	<0.001
Physical QoL	51.7 ± 10.1	52.3 ± 7.6	−0.1 (−0.5, 0.4)	0.763	0.576	0.613
Mental QoL	38.6 ± 9.0	47.7 ± 9.5	−1.0 (−1.5, −0.5)	<0.001	<0.001	<0.001

Model 1: adjusted for age and sex; Model 2: adjusted for age, sex, deafness at birth or later, hearing aids used, and language used.

**Table 4:** Unadjusted and adjusted differences in mental disorders (depression, anxiety, and stress) and quality of life (physical and mental) according to suicide risk in the post-COVID-19 period, analyzed using Student’s *t* test and ANCOVA in men and women.

	Men					
Variable	Suicide Risk (*n* = 22)	No Suicide Risk (*n* = 79)	Cohen’s *d* (95% CIs)	Unadjusted Model	Model 1	Model 2
Depression	10.1 ± 4.2	3.5 ± 3.1	2.0 (1.4, 2.5)	<0.001	<0.001	<0.001
Anxiety	7.7 ± 4.5	2.4 ± 2.4	1.8 (1.3, 2.3)	<0.001	<0.001	<0.001
Stress	9.9 ± 3.9	4.9 ± 4.5	1.1 (0.6, 1.6)	0.004	0.006	0.011
Physical QoL	54.3 ± 6.1	52.3 ± 8.2	0.3 (−0.2, 0.7)	0.502	0.512	0.407
Mental QoL	42.3 ± 9.1	47.9 ± 9.5	−0.6 (−1.1, −0.1)	0.117	0.137	0.197
	Women					
Depression	9.1 ± 5.2	4.1 ± 4.4	1.1 (0.6, 1.6)	<0.001	0.002	0.003
Anxiety	7.4 ± 5.9	2.7 ± 3.4	1.2 (0.7, 1.7)	<0.001	<0.001	<0.001
Stress	11.0 ± 5.3	5.4 ± 3.9	1.3 (0.8, 1.8)	<0.001	<0.001	<0.001
Physical QoL	49.9 ± 12.1	52.3 ± 7.2	−0.3 (−0.8, 0.2)	0.381	0.155	0.237
Mental QoL	36.1 ± 8.4	47.5 ± 9.7	−1.2 (−1.7, −0.7)	<0.001	<0.001	0.001

Model 1: adjusted for age; Model 2: adjusted for age, deafness at birth or later, hearing aids used, and language used.

## Data Availability

The data are available in the manuscript or by contacting the corresponding author.

## Supplementary Materials

The following supporting information can be downloaded at: https://www.mdpi.com/article/10.3390/jcm14093130/s1, Table S1: Centres and associations for the hearing impaired in Spain included in the study; Table S2: SF-12 Health Questionnaire; Table S3: Depression, Anxiety and Stress Scale (DASS-21); Table S4: Plutchik’s Suicide Risk Scale.

## Abbreviations

COVID-19	Coronavirus disease 2019
HI	Hearing impairment
PPE	Personal protective equipment
QoL	Quality of life
SLI	Sign language interpreter
WHO	World Health Organization
