# Supplementary material for: Suicide Risk in People with Hearing Impairment in the Post-COVID-19 Period: The CaViDAuCo Study
Source: J Clin Med. 2025 Apr 30;14(9):3130. doi: 10.3390/jcm14093130 (PMC12072992; doi:10.3390/jcm14093130)
Supplement: Supplementary file 1 [file jcm-14-03130-s001.zip › jcm-3600692-supplementary.pdf]

SUPPLEMENTARY MATERIAL

Table S1. Centres and associations for the hearing impaired in Spain included in the study.

**Centres/Associations of people with HI**	**Province (city) or autonomous community**
ACOPROS (Association for Collaboration and Promotion of the Deaf in A Coruña)	A Coruña
ASPAS Albacete (Parents and Friends of the Deaf Association of Albacete)	Albacete
APANAS Aspe (Association of Parents and Friends of Deaf Children and Adolescents of Aspe)	Alicante
APANAH (Association of Parents and Hearing Loss People of Alicante)	
UNASORD (Andalucía Union of Organizations for Deaf People)	Andalucía
AFA (Andalucía Foundation for Accessibility and Deaf People)	
ASZA (Association of Deaf People of Zaragoza and Aragon)	Aragón
FAAPAS (Aragonese Federation of Associations of Parents and Friends of People affected by Deafness)	
APADA (Association of Parents and Friends of People with Hearing Impairment of Asturias)	Asturias
FESOPRAS (Federation of Deaf People of the Principality of Asturias)	
ADABA (Association of People with Hearing Impairment of Badajoz)	Badajoz
ARANSBUR (Association of Families of Deaf People of Burgos)	Burgos
ASCAPAS (Association of Parents and Friends of the Deaf of Cáceres)	Cáceres
FESCAN (Federation of Associations of Deaf People of Cantabria)	Cantabria
ASPAS Castellón (Association of Parents and Friends of the Deaf of Castellón)	Castellón
FESORMANCHA (Federation of Deaf People of Castilla-La Mancha)	Castilla-La Mancha
FAPSCL (Federation of Associations of Deaf People of Castilla and León)	Castilla and León
ACAPPS (Federation of Catalan associations of parents and deaf people)	Cataluña
FESOCA (Federation of Deaf People of Cataluña)	
ASPAS Ciudad Real (Association of Parents and Friends of the Deaf of Ciudad Real)	Ciudad Real
ASPAS Córdoba (Parents and Friends of the Deaf Association of Córdoba)	Córdoba
ASPAS Cuenca (Parents and Friends of the Deaf Association of Cuenca)	Cuenca
ACSOC (Cuenca Deaf Cultural Association)	
FEXAS (Extremadura Federation of Associations of Deaf People)	Extremadura
FAXPG (Federation of Associations of Deaf People of Galicia)	Galicia
ASPRODES Granada (Association for the Rights of Deaf People of Granada)	Granada
APANDAGU (Association of Parents and Friends of Children with Hearing Loss of Guadalajara)	Guadalajara
APSORGU (Cultural Association of Deaf People of Guadalajara)	
ASPRODESORDOS Huelva (Parents and Friends of the Deaf Association of Huelva)	Huelva
San Francisco de Sales Association for the Hard of Hearing	Huesca
FSIB (Federation of Deaf People of the Balearic Islands)	Islas Baleares
FASICAN (Federation of Associations of Deaf People of the Islas Canarias)	Islas Canarias
AFAIS Jaén (Association of Families and Friends of Deaf Children of Jaén)	Jaén
APROSOJA (Provincial Association of Deaf People of Jaén)	
FUNCASOR La Palma (Canary Islands Foundation for People with Deafness)	La Palma
ASR (Association of Deaf People of La Rioja)	La Rioja

ASFAS León (Association of Family and Friends of the Deaf of León)	León
ABIPANS (Bilingual Association of Parents of Deaf Children of the Community of Madrid)	Madrid
ASPAS Madrid (Parents and Friends of the Deaf Association of Madrid)	
FESORCAM (Federation of Deaf People of the Community of Madrid)	
ASPANSOR Málaga (Association of Parents and Friends of the Deaf of Málaga)	Málaga
ASPAS Mallorca (Association of Parents and Friends of the Deaf of Mallorca)	Mallorca
ASOME (Association of Deaf People of Melilla)	Melilla
ASPANPAL Murcia (Association of Parents of Children with Hearing and Language Problems of Murcia)	Murcia
APANDA Cartagena (Association of Parents of Children with Hearing Impairments of Cartagena)	
FESORMU (Federation of Deaf People of the Region of Murcia)	
EUNATE (Association of Families of People with Hearing Impairments of Navarra)	Navarra
ASORNA (Association of Deaf People of Navarra)	
Euskal Gorrak (Federation of Associations of Deaf People of País Vasco)	País Vasco
ASPAS Salamanca (Association of Parents of Deaf Children of Salamanca)	Salamanca
ASPAS Sevilla (Association of Families and Friends of Deaf People of Sevilla)	Sevilla
ATPANSOR (Teruel Association of Parents and Friends of Deaf Children)	Teruel
APANDAPT (Association of Parents and Friends of Children with Hearing Loss of Toledo)	Toledo
ASPAS Valencia (Association of Families and Deaf People of Valencia)	Valencia
FESORD (Federation of Deaf People of the Valencia Community)	
ASPAS Valladolid (Association of Parents and Friends of the Deaf of Valladolid)	Valladolid
ASPANSOR Zaragoza (Association of Parents of Deaf Children of Zaragoza)	Zaragoza

*HI = hearing impairment

Table S2. SF-12 Health Questionnaire.

Item	Question	Response options
**1**	In general, would you say your health is:	Excellent / Very good / Good / Fair / Poor
**2**	Moderate activities such as moving a table, pushing a vacuum cleaner, bowling, or playing golf: Does your health now limit you?	YES, limited a lot / YES, limited a little / NO, not limited at all
**3**	Climbing several flights of stairs: Does your health now limit you?	YES, limited a lot / YES, limited a little / NO, not limited at all
**4**	During the past 4 weeks, have you accomplished less than you would like as a result of your physical health?	Yes / No
**5**	During the past 4 weeks, were you limited in the kind of work or other activities due to your physical health?	Yes / No
**6**	During the past 4 weeks, have you accomplished less than you would like due to emotional problems (such as feeling depressed or anxious)?	Yes / No
**7**	During the past 4 weeks, did you work or do other activities less carefully than usual due to emotional problems (such as feeling depressed or anxious)?	Yes / No
**8**	During the past 4 weeks, how much did pain interfere with your normal work (including work outside the home and housework)?	Not at all / A little bit / Moderately / Quite a bit / Extremely
**9**	During the past 4 weeks, how much of the time have you felt calm and peaceful?	All of the time / Most of the time / A good bit of the time / Some of the time / A little of the time / None of the time
**10**	During the past 4 weeks, how much of the time during did you have a lot of energy?	All of the time / Most of the time / A good bit of the time / Some of the time / A little of the time / None of the time
**11**	During the past 4 weeks, how much of the time have you felt down-hearted and blue?	All of the time / Most of the time / A good bit of the time / Some of the time / A little of the time / None of the time
**12**	During the past 4 weeks, how much of the time has your physical health or emotional problems interfered with your social activities (like visiting friends, relatives, etc.)?	All of the time / Most of the time / Some of the time / A little of the time / None of the time

Assessment: the responses obtained were analyzed individually through the SF12 OrthoToolKit calculator [28], which calculates the scores of the physical and mental level of each subject.

Table S3. Depression, Anxiety and Stress Scale (DASS-21).

**1 (s)**	I found it hard to wind down	0	1	2	3
**2 (a)**	I was aware of dryness of my mouth	0	1	2	3
**3 (d)**	I couldn't seem to experience any positive feeling at all	0	1	2	3
**4 (a)**	I experienced breathing difficulty (e.g. excessively rapid breathing, breathlessness in the absence of physical exertion)	0	1	2	3
**5 (d)**	I found it difficult to work up the initiative to do things	0	1	2	3
**6 (s)**	I tended to over-react to situations	0	1	2	3
**7 (a)**	I experienced trembling (e.g. in the hands)	0	1	2	3
**8 (s)**	I felt that I was using a lot of nervous energy	0	1	2	3
**9 (a)**	I was worried about situations in which I might panic and make a fool of myself	0	1	2	3
**10 (d)**	I felt that I had nothing to look forward to	0	1	2	3
**11 (s)**	I found myself getting agitated	0	1	2	3
**12 (s)**	I found it difficult to relax	0	1	2	3
**13 (d)**	I felt down-hearted and blue	0	1	2	3
**14 (s)**	I was intolerant of anything that kept me from getting on with what I was doing	0	1	2	3
**15 (a)**	I felt I was close to panic	0	1	2	3
**16 (d)**	I was unable to become enthusiastic about anything	0	1	2	3
**17 (d)**	I felt I wasn't worth much as a person	0	1	2	3
**18 (s)**	I felt that I was rather touchy	0	1	2	3
**19 (a)**	I was aware of the action of my heart in the absence of physical exertion (e.g. sense of heart rate increase, heart missing a beat)	0	1	2	3
**20 (a)**	I felt scared without any good reason	0	1	2	3
**21 (d)**	I felt that life was meaningless	0	1	2	3

*(a) = anxiety; (d) = depression; (s) = stress.

Assessment: item scores (subscales) for depression (items 3, 5, 10, 13, 16, 17, and 21), anxiety (items 2, 4, 7, 9, 15, 19, and 20), and stress (items 1, 6, 8, 11, 12, 14, and 18) were interpreted individually. For each subscale, the scores of the corresponding items are summed, and the result is then multiplied by two. The final total score was classified according to the following cut-off points to assess the degree of symptomatology [29]:

	**Normal**	**Mild**	**Moderate**	**Severe**	**Extremely severe**
**Depression**	0–9	10–13	14–20	21–27	28+
**Anxiety**	0–7	8–9	10–14	15–19	20+
**Stress**	0–14	15–18	19–25	26–33	34+

Table S4. Plutchik's Suicide Risk Scale.

**1.** Do you take drugs such as aspirins or sleeping pill regularly?	Yes	No
**2.** Do you have trouble falling asleep?	Yes	No
**3.** Do you sometimes fear that you will lose control of yourself?	Yes	No
**4.** Do you have little interest in being with people?	Yes	No
**5.** Do you see your future more pessimistic than optimistic?	Yes	No
**6**. Do you ever feel that you are worthless?	Yes	No
**7.** Do you feel hopeless about the future?	Yes	No
**8.** Do you often feel so frustrated that you just want to lie down and quit struggling altogether?	Yes	No
**9.** Do you feel depressed now?	Yes	No
**10.** Are you separated, divorced, or widowed?	Yes	No
**11.** Has anyone in your family ever tried to commit suicide?	Yes	No
**12.** Have you ever been so angry that you felt you might kill someone?	Yes	No
**13.** Have you ever thought about committing suicide?	Yes	No
**14.** Have you ever told anyone that you would commit suicide?	Yes	No
**15.** Have you ever tried to take your life?	Yes	No
**Total Score**		

Assessment: with each affirmative response receiving 1 point and a negative response receiving 0 points, for a total possible score between 0 and 15 points. As the scale score increases, so does the suicide risk, establishing a cut-off point greater than or equal to 6 to consider the suicide risk to be significant [30].
